# Impact of Breast Density Awareness on Knowledge about Breast Cancer Risk Factors and the Self-Perceived Risk of Breast Cancer

**DOI:** 10.3390/diagnostics10070496

**Published:** 2020-07-20

**Authors:** Kristina Bojanic, Sonja Vukadin, Filip Sarcevic, Luka Malenica, Kaja Grgic, Robert Smolic, Kristina Kralik, Ines Bilic Curcic, Gordana Ivanac, George Y. Wu, Martina Smolic

**Affiliations:** 1 Department of Biophysics and Radiology, Faculty of Dental Medicine and Health Osijek, J.J. Strossmayer University of Osijek, 31000 Osijek, Croatia; kristina.bojanic@dzo.hr; 2 Department of Biophysics and Radiology, Faculty of Medicine Osijek, J.J. Strossmayer University of Osijek, 31000 Osijek, Croatia; 3 Department of Radiology, Health Center Osijek, 31000 Osijek, Croatia; 4 Department of Pharmacology and Biochemistry, Faculty of Dental Medicine and Health Osijek, J.J. Strossmayer University of Osijek, 31000 Osijek, Croatia; sonya.sarcevic@gmail.com; 5 Department of Pharmacology, Faculty of Medicine Osijek, J.J. Strossmayer University of Osijek, 31000 Osijek, Croatia; filip.sarcevic@gmail.com (F.S.); kaja.grgich@gmail.com (K.G.); ibcurcic@mefos.hr (I.B.C.); 6 Department of Pathophysiology, Physiology and Immunology, Faculty of Dental Medicine and Health Osijek, J.J. Strossmayer University of Osijek, 31000 Osijek, Croatia; luka.malenica@fdmz.hr (L.M.); robert.smolic@fdmz.hr (R.S.); 7 Department of Pathophysiology, Faculty of Medicine Osijek, J.J. Strossmayer University of Osijek, 31000 Osijek, Croatia; 8 Department of Internal Medicine, University Hospital Osijek, 31000 Osijek, Croatia; 9 Department of Medical Statistics and Medical Informatics, Faculty of Medicine Osijek, J.J. Strossmayer University of Osijek, 31000 Osijek, Croatia; kristina.kralik@mefos.hr; 10 Department of Diagnostic and Interventional Radiology, University Hospital Dubrava, 10000 Zagreb, Croatia; gordana.augustan@gmail.com; 11 Department of Medicine, Division of Gastroenterology/Hepatology, University of Connecticut Health Center, 263 Farmington Avenue, Farmington, CT 06032, USA; wu@uchc.edu

**Keywords:** breast density, breast cancer, risk-based screening, awareness, knowledge, breast cancer risk perception, health literacy

## Abstract

Breast density (BD) reduces sensitivity of mammography, and is a strong risk factor for breast cancer (BC). Data about women’s awareness and knowledge of BD are limited. Our aim is to examine whether the BD information disclosure and BD awareness among women without BC are related to their knowledge about BC risk factors. We examined self-reported BC risk perception and its association to BD awareness and level of health literacy. A cross-sectional, single site study included 263 Croatian women without BC who had mammographic examination. Data were collected by interviews using questionnaires and a validated survey. Of the total, 77.1% had never heard of BD, and 22.9% were aware of their BD. Most participants who knew their BD (88.2%, *p* < 0.001) had higher levels of education. Majority of subjects (66.8%) had non-dense breasts and 33.2% had dense breasts. Subjects aware of their BD knew that post-menopausal hormone replacement therapy (*p* = 0.04) and higher BD (*p* = 0.03) are BC risk factors. They could more easily access information about health promotion (*p* = 0.03). High-BD informed women assessed their lifetime BC risk as significantly higher than all others (*p* = 0.03). Comprehension of BD awareness and knowledge is crucial for reinforcement of educational strategies and development of amendatory BC screening decisions.

## 1. Introduction

Breast cancer (BC), as the most prevalent cancer in women and the second leading cause of female cancer deaths, persists in being accounted for a major health and socioeconomic burden. The latest release of the Global Cancer Observatory by the International Agency for Research on Cancer estimates that BC incidence and mortality rate will continue to rise over the next 20 years, with over 3 million patients and 992,000 deaths from BC in 2040 [1]. The magnitude of the problem is represented by the fact that every 18 seconds one woman is diagnosed with BC, and every eighth woman carries the risk of getting this malignant disease [2]. Currently, the most effective strategy for reducing BC mortality is early detection through mammographic screening [3].

Breast density (BD) denotes the mammographic appearance of the breast, with different proportions of fibroglandular and fatty tissue as the basic components of breast tissue [4]. Although there are several tools to assess BD, the most commonly used in clinical practice is the qualitative one from the American College of Radiology (ACR) Breast Imaging Reporting and Data System (BI-RADS) [5]. According to the fifth edition of the ACR BI-RADS Atlas, BD is subjectively estimated by the radiologist as the relative amount of radiopaque fibroglandular tissue in relation to radiolucent fatty tissue [5,6]. It defines four categories of BD, including type A: almost entirely fatty; type B: scattered fibroglandular densities; type C: heterogeneously dense; and type D: extremely dense [5,6]. Although two-dimensional measure, subjective assessment, and inter- and intra-reader variability in radiologists’ interpretation of breast density [7] limit the accuracy of the ACR BI-RADS BD estimation, women with BD types C and D are considered to have mammographically dense breasts [4].

Mammographically dense breasts have been associated with a masking effect on mammography, since dense tissue can obscure cancers, thus lowering the sensitivity of mammography [8,9]. Apart from the fact that BD is one of the key factors for false-negative mammography findings, it is also associated with a three-fold increase in recall rate and false-positive mammography findings, thus decreasing its specificity [6,10]. More importantly, dense breasts have been identified as a strong and independent risk factor for the development of BC [11,12,13]. Literature data confirm that women with BD type D have a four- to six-fold increase in risk compared with those with BD type A [14,15]. Although age and genetic mutations (*BRCA 1/BRCA2*) are the strongest risk factors for BC, BD is more common in the general population [6]. About 50% of women between 40–74 years in the United States have dense breasts on mammography [13,16,17]. Thus, some studies suggested that increased BD alone contributes to a significant proportion of cancer risk at the population level, accounting for 16% of all BC diagnosed [11].

Although BD reduces the sensitivity of mammography and is an independent risk factor for BC, physicians in countries of the European Union (EU) are not obligated to notify women about it. In contrast, the U.S. Congress in 2019 passed a national BD notification law, and the U.S. Food and Drug Administration (FDA) proposed that mammogram centers must inform women if they have dense breasts. Since 2009, when BD information legislation passed in Connecticut as the first state in the United States with a BD notification law, there was growing evidence supporting the initiative of BD legislation as an effective tool in increasing knowledge of BD, which further impacts BC screening [18]. Still, the current literature on women’s awareness and knowledge of BD is limited [19,20], with conflicting results [3,19]. Also, mixed results have been reported in the literature about the association between level of health literacy and BC detection in different ethnic and racial groups [21,22].

Comprehension of the prevailing state of BD awareness and knowledge is crucial for the reinforcement of educational strategies and development of amendatory BC screening decisions.

The aim of this study was to examine whether the BD information disclosure and BD awareness among Croatian women without BC are related to their knowledge about BC risk factors. Additionally, we examined self-reported BC risk perception and its association to BD awareness and the level of health literacy in the same group of participants.

## 2. Participants and Methods

### 2.1. Subject Population

This was a single-center, cross-sectional prospective study involving 263 women without BC who were referred for a mammographic examination at the Osijek Health Center Breast Cancer Clinic. Participants were included successively during a one-year period if they agreed to participate in the study and did not meet the exclusion criteria. The exclusion criteria included personal history of invasive BC; lobular carcinoma in situ (LCIS) or ductal carcinoma in situ (DCIS); positive result of genetic testing of the *BRCA1*/*BRCA2* gene; diagnosis of hereditary cancer-related syndrome based on genetic testing; and previous radiotherapy treatment of the thorax.

Ethical approval for this study was obtained from the Health Center Osijek Review Board (approval number: 03-319-1/19). All research involving human subjects and material derived from human subjects in this study was done in accordance with ethical principles outlined in the World Medical Association Declaration of Helsinki–Ethical Principles for Medical Research Involving Human Subjects (initiated in June 1964, last amendment in October 2000). All participants signed an informed consent form before being included in the study.

### 2.2. Study Design and Procedure

Radiologic evaluation of BD was performed by one very experienced breast radiologist, with over 10 years’ experience in breast radiology; thus, “interobserver” variability was excluded. The fifth edition of ACR BI-RADS Atlas was used for the purpose of qualitative estimation of BD. All women underwent digital mammography on a dedicated mammography machine (Amulet, Shimadzu, Kyoto, Japan).

All data were collected by face-to-face interviews, conducted by three trained sixth-year medical school students. A 30-item questionnaire, divided into three sections, was developed for the purpose of the study. The first part of the questionnaire examined the participant history and sociodemographic data, disclosure of BD information, and BD awareness. The second part examined knowledge about the BC risk factors through 16 questions. The third part examined self-perceived BC risk and concerns about possible BC diagnosis.

BD data were disclosed to women if a radiologist or health care provider had informed women about their own BD.

BD awareness was considered to be established (defined as positive) if the women had been notified about their BD and were able to recall the disclosed BD.

BD knowledge was defined as positive if women knew about the association between high BD and an increased risk of developing BC [16].

A validated European Health Literacy (HLS) survey EU-Q47 (HLS-EU-Q47) was administered to evaluate the level of health literacy among participants. Permission to use HLS-EU-Q47 was granted by the HLS EU Consortium. It contains 47 items examining subject competence in accessing, understanding, appraising, and applying health-related information within three domains: healthcare, disease prevention, and health promotion. Each question was graded using a five-point Likert scale (i.e., 1 = very difficult, 2 = difficult, 3 = easy, 4 = very easy, and 5 = don’t know—used only by the examiner) [23]. The questionnaire was forward-translated from English into the Croatian language, and back-translated from Croatian into English by two independent translators, and minor changes were done to ensure that wording was appropriate for the local context.

### 2.3. Statistical Analysis

Categorical data were represented by absolute and relative frequencies. Numerical data were described by the median and the limits of the interquartile range. Differences of categorical variables were tested by Chi-square test and by Fisher’s exact test. The normality of the distribution of numerical variables was tested by the Shapiro–Wilk test. Differences between two independent groups were tested by Mann–Whitney’s *U* test. The significance level was set to alpha = 0.05. MedCalc Statistical Software version 19.1.7 (MedCalc Software Ltd., Ostend, Belgium) was used for statistical analysis [24].

## 3. Results

### 3.1. Women’s Demographic Characteristics, BD Data Disclosure and Differences in BD Awareness

A total of 263 subjects were included in this cross-sectional study, with a median age of 58 years (interquartile range (IQR) 25–75% = 46.5–62.0).

BD information was disclosed to 65 (25%) participants only and was not disclosed to 157 (60%) participants, while 41 (15%) participants were not sure whether they had received their BD notification.

When asked about the awareness of BD, 172 (77.1%) out of a total of 263 subjects had never heard of the term, 51 (22.9%) were aware of their BD, and the remaining 40 did not know how to answer the question. The latter were excluded from further analysis. It is interesting that out of 172 women who were not aware of their BD, 20 (11.7%) were sure that BD information had been disclosed to them by radiologists or health care providers. However, they could not recall whether the disclosed information was related to high or low BD (Table 1, second row).

Of the remaining 223 women, according to the level of education, a majority of women had a high school diploma (62%), and 15% had a master’s degree. With regards to employment status, 38% of the participants were retired at the time of enrollment in the study, and 28% were working in the public sector. Of the total subjects, 17.5% had first-degree relatives with BC, and 52.3% had any type of cancer in their family. Most of the subjects were menopausal (75%), with a median of two live births. Median BMI was 25.9, with an IQR of 24.1-29.3. More than half of the women (55.6%) suffer from some chronic illness for which daily therapy is required, but less than a quarter smoke cigarettes (21.5%), and only 1% consumes alcohol regularly (Table 1; third column, “Total”).

Significantly more participants who were notified about BD knew their actual BD (88.2%, *p* < 0.001). Women aware of their BD had a higher level of education (29.4%; *p* = 0.01). There were no statistical differences in other historical and sociodemographic data with regards to awareness of BD information (Table 1).

### 3.2. Radiological Evaluation of BD

Based on the radiological evaluation of breast density, the participants were first divided into four groups: ACR type A 76 (28.9%), ACR type B 83 (31.6%), ACR type C 54 (20.5%), and ACR type D 30 (11.4%). For 20 subjects (7.6%), radiological density assessment was not performed or recorded due to different technical reasons. Finally, a total of 211 subjects were divided into two groups. The non-dense-breast group included women with ACR BD types A and B, with 66.8% of participants. Women with BD types C and D were included in a dense breast group, with 33.2% of participants (Table 2; first three rows in the third column “Total”).

In the group of women informed about their BD, there were no statistically significant differences in BD awareness with regard to the radiologically estimated BD (Table 2; seventh to ninth rows). Also, in the group of women who were not informed about their BD, there were no statistically significant differences in BD awareness with regard to the radiological assessment of BD (Table 2; sixth to ninth row).

Subjects with mammographically dense breasts based on radiological analysis were statistically significantly younger, had a lower BMI, and a lower interquartile range of full-term pregnancies (Table 3).

### 3.3. Knowledge about BC Risk Factors and BC Risk Perception According to BD Awareness

**Figure 1:**
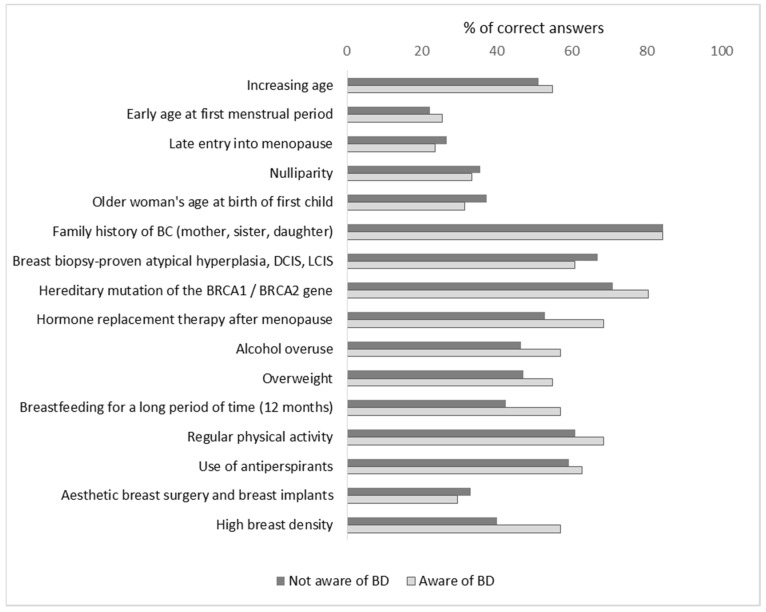
Breast cancer (BC) risk factor knowledge with regard to BD awareness. Patients aware of their BD knew significantly more frequently that taking the hormone replacement therapy after menopause (*p* = 0.04) and higher BD (*p* = 0.03) are BC risk factors.

The overall knowledge about BC risk factors was averaged, with a median of eight correct answers (IQR 6–10) out of a total of 16 questions. The most widely known risk factor was the first-degree relationship with a person who had BC (mother, sister, daughter, grandmother), which was accurately recognized by 84.3% of participants (Figure 1, sixth row). Patients aware of their BD knew that taking hormone replacement therapy after menopause (*p* = 0.04) and higher breast density (*p* = 0.03) are BC risk factors (Figure 1, 9th and 16th rows). However, there were no other statistically significant disparities in BC knowledge between the groups with respect to BD data awareness.

There were no differences in BC risk perception or concerns about possible BC diagnosis with regard to BD awareness when the entire group of participants was analyzed (Table 4; first six rows). Additionally, the subgroup of women who were informed by radiologists about the high density of their breasts assessed their lifetime BC risk as significantly higher compared to all other subjects (*p* = 0.03). However, that subgroup of women had no statistically significant differences in concerns about possible BC compared to all other subjects.

### 3.4. Health Literacy

**Figure 2:**
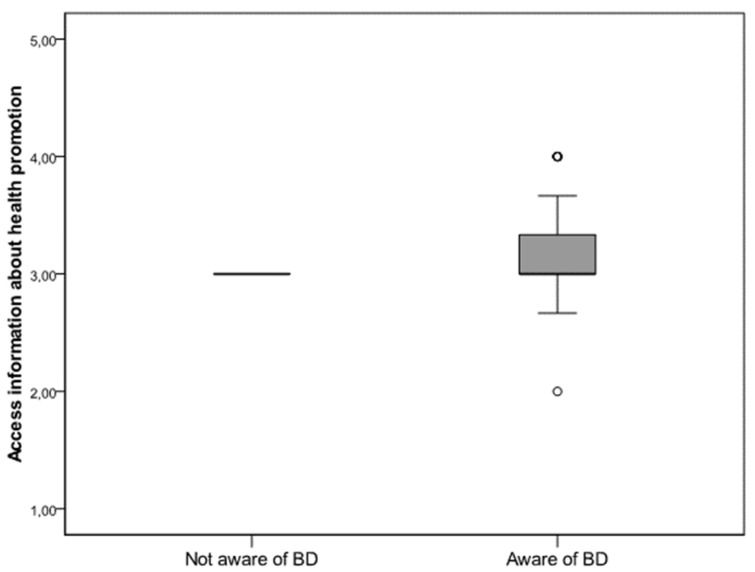
Level of health literacy in regard to BD awareness. Patients aware of their BD could access information about health promotion more easily based on validated HLS-EU-Q47 questionnaires (Mann–Whitney *U* test; *p* = 0.03).

Participants who were aware of their BD could more easily access information within the health promotion domain based on HLS-EU-Q47 (*p* = 0.03) (Figure 2). No statistically significant differences were found between women who were aware of their BD and those who were not aware of it, with regard to the remaining domains of the questionnaire.

## 4. Discussion

In this cross-sectional study, awareness of BD among women without BC and knowledge about BD as a BC risk factor were examined. In study by Katapodi et al., it was demonstrated that BC risk awareness can positively influence BC screening rates [25]. Therefore, we hypothesized that BD information disclosure and BD awareness could be major contributing factors in general knowledge about BC risk factors, and ultimately might influence women’s interest in different prevention strategies.

In the current study, BD information was disclosed to 25% of subjects, but only 23% of the women were aware of their BD, which is significantly lower compared to the recent, still scarce literature data [18,19,26] representing the U.S. population after mandatory BD information disclosure. Our results are somewhat in line with studies conducted when BD reporting was not obligatory in United States [27], and with low cancer risk factors awareness in general in low- and middle-income countries [28]. To our knowledge, this is the first study of this kind conducted in the territory of the European Union. The obtained results are in line with our expectations, since in the countries of the European Union it is not obligatory to inform women about the density of their breasts after the mammography examination. Thus, there is no reason for women to have a better awareness of BD after initial participation in the study.

Out of 172 women not aware of their BD, 20 (11.7%) had BD information disclosed to them by radiologists or health care providers. However, they did not adequately understand the obtained information. This paradox has been confirmed by other research groups, suggesting that just informing women about BD does not necessarily mean correct understanding of the disclosed information [19]. Authors concluded that merely notifying a woman about her BD is not enough, and the BD information disclosure should be understood as the first step in wider educational campaign about the real significance of BD in women’s health.

Our research confirmed that women aware of their BD had a higher level of education. Similar results have been obtained in several previous studies, implying that the woman’s level of education is a strong predictor of her knowledge, with BD awareness strongly associated with a higher level of education [18,23].

Data related to the radiological assessment of BD were in line with expectations, with 33% of women having mammographically dense breasts, which is slightly less in comparison with studies carried out in the United States, like the study of Lee et al., which had about 43% women with dense breasts [6]. Possible explanations for these slight disparities could be a very small proportion of women in Croatia who consume hormone replacement therapy after menopause (which has been shown to be associated with increasing BD [29]) and a higher proportion of overweight women in our study (median BMI of our study population was 25.9). Subjects with mammographically dense breasts based on radiological analysis were significantly younger, had a lower BMI, and a lower interquartile range of full-term pregnancies. The obtained results are consistent with previous studies about the inverse association of age, BMI, and the number of full-term pregnancies with BD [30,31].

The overall knowledge of BC risk factors was moderate, which was surprisingly above expectations given the low percentage of BD disclosure and awareness among the women surveyed. A minority (44%) of women knew that higher breast density was a risk factor for BC, significantly more those aware of their BD. Comparison of our results with other previously published research is limited by the inconsistency of the definition of BD awareness and knowledge, which significantly differ between studies [18,19,27,32,33]. Also, these disparities may be attributed to study design and sampling methodology. One explanation for our results could be that we examined general knowledge about BC risk factors through 16 questions, including a number of other BC risk factors, besides BD. In the future, the research focus should be on BD, with an extension of the question in terms of knowing that higher BD increases the risk for BC and reduces the sensitivity of mammography to detect BC. We can assume that this knowledge would be significantly lower, since there is no legal obligation to inform women about the density of their breasts in Croatia. In addition, it is worth mentioning that disclosure of BD has certain shortcomings observed in recent studies—i.e., a study conducted in Massachusetts (United States) confirmed that the negative consequences of mandatory BD disclosure include misinformation and confusion regarding knowledge and further women’s health procedures [32]. A similar study conducted in Virginia suggested that density notification laws have a positive effect on the level of awareness of BD. However, the link with knowledge was lacking [19].

A very interesting result of the current study is that informed women aware of their higher BD assessed their lifetime BC risk as significantly higher compared to all other subjects. This result represents the goal of BD notification legalization, with expected further changes in breast health care decision making, allowing women to make personalized screening choices based on their risk.

Women aware of their BD could more easily assess information within the health promotion domain based on the HLS-EU-Q47. In the current surveyed group of women, this subgroup had a higher level of education, which is consistent with literature data [22,34,35]. However, further research should clarify this result in more detail, and elucidate whether a higher level of health literacy could be a predictor of better BD awareness. Nevertheless, this result strongly suggests that there is a pronounced need for educational programs and materials, which will generally contribute to raising the level of women’s health awareness.

The current study has a few limitations. In our group of subjects, there were no differences in race or ethnicity, since all included subjects were Caucasian, thus limiting the ability to generalize to the entire population. Additionally, the questionnaires were distributed in only one radiology center, representing only a subset of the general population. Small sample size is another limiting factor. However, a great advantage of our research is that it was conducted as face to-face interviews by three trained sixth-year medical school students, available to constantly provide clarification and answer any questions. The vast majority of research published so far on this issue was conducted through an anonymous survey sent by mail or as a telephone call. The main strength of our research is proving the existence of low level of BD information disclosure and awareness among women in Croatia. The idea is to position radiologists and primary health care providers as principal representatives in BD information disclosure, raising BD awareness, and knowledge among women.

## 5. Conclusions

The results of the current study show that many women in Croatia are not familiar with the term BD. It is just one but one of the most prevalent factors that should be considered when women discuss risk-based BC screening options with healthcare providers. Digital mammography remains the primary screening tool for women with dense breasts. In the current study, BD information was disclosed to only one quarter of participants. However, 88% of the notified participants were able to comprehend the notification. These women also had a higher level of health literacy. BD information awareness was associated with significantly better knowledge of some BC risk factors and higher assessments of lifetime BC risk. Results from this study provide valuable information for legislation of BD data in Croatia.

## Figures and Tables

**Table 1:** Historical and sociodemographic data.

BD Awareness (*n* (%))		Not Aware of BD (*n* = 172 (77.1%))	Aware of BD (*n* = 51 (22.9%))	Total (*n* = 223)	*p*
BD information disclosed (*n* (%))		20 (11.7)	45 (88.2)	65 (29.3)	<0.001 *
Age (Median (25–75%))		58 (49–63)	57 (49–63)	58 (46.5–62)	0.91 ^§^
BMI (Median (25–75%))		26.6 (24.4–30.1)	25.1 (23.3–29.1)	25.9 (24.1–29.3)	0.11 ^§^
Live birth (Median (25–75%))		2 (1–2)	2 (1–2)	2 (1–2)	0.94 ^§^
Education status (*n* (%))					
	Primary School Diploma	29 (16.9)	4 (7.8)	33 (14.8)	0.01 *
	High School Diploma	111 (64.5)	28 (54.9)	144 (60)	
	Bachelor’s Degree	13 (7.6)	4 (7.8)	17 (7.6)	
	Master’s Degree	19 (11)	15 (29.4)	34 (15.2)	
	Doctorate	0	0	0	
Work status (*n* (%))					
	Pupil	1 (0.6)	0	1 (0.4)	0.08 *
	Student	1 (0.6)	0	1 (0.4)	
	Private Sector Employee	28 (16.3)	6 (11.8)	34 (15.2)	
	Public Sector Employee	42 (24.4)	20 (39.2)	62 (27.8)	
	Free profession	2 (1.2)	1 (2)	3 (1.3)	
	Unemployed	35 (20.3)	3 (5.9)	38 (17)	
	Retired	63 (37)	21 (41)	84 (38)	
Any type of cancer in first degree relative (*n* (%))		88 (51.5)	28 (54.9)	116 (52.3)	0.67 *
Family history of BC (*n* (%))		30 (17.4)	9 (17.6)	39 (17.5)	0.97 *
An active menstrual cycle (*n* (%))		44 (25.6)	11 (21.6)	55 (24.7)	0.24 *
Suffers from chronic illness for which daily therapy is required (*n* (%))		97 (56.4)	27 (52.9)	124 (55.6)	0.66 *
Cigarette smoker (*n* (%))		37 (21.5)	11 (21.6)	48 (21.5)	0.99 *
Regularly consumes alcoholic beverages ** (*n* (%))		2 (1.2)	0	2 (0.9)	>0.99 *

^§^ Mann–Whitney *U* test; * χ^2^ test.

**Table 2:** Radiological evaluation of breast density and differences in breast density (BD) awareness according to BD information disclosure.

BD Awareness		Number (%)			*p* *
		Not Aware of BD	Aware of BD	Total	
ACR BI-RADS					
	Non-dense breast	114 (69.9)	27 (56.3)	141 (66.8)	0.08
	Dense breast	49 (30.1)	21 (43.8)	70 (33.2)	
	Total	163 (100)	48 (100)	211 (100)	
BD information not disclosed					
	Women with non-dense breast	103 (72.5)	5 (83.3)	108 (73)	>0.99
	Women with dense breast	39 (27.5)	1 (16.7)	40 (27)	
	Total	142 (100)	6 (100)	148 (100)	
BD information disclosed					
	Women with non-dense breast	11 (55)	22 (52.4)	33 (53.2)	>0.099
	Women with dense breast	9 (45)	20 (47.6)	29 (46.8)	
	Total	20 (100)	42 (100)	62 (100)	

* Fisher’s Exact test.

**Table 3:** Differences in data regarding the radiological assessment of BD.

	Median (25–75%) in Regard to Radiological Assessment of BD			*p* *
	Non-Dense Breast	Dense Breast	Total	
Age	59 (54–63)	47 (39–58)	58 (46.5–62)	<0.001
BMI	27.5 (25.1–31.2)	24.4 (22.1–23.0)	25.9 (24.1–29.3)	<0.001
Full-term pregnancies	2 (2–2)	2 (1–2)	2 (1–2)	0.003

* Mann–Whitney *U* test.

**Table 4:** Comparison of self-assessed estimates of BC risks and self-assessed estimates of worry about BC development with regards to BD awareness.

	Not Aware of BD	Aware of BD	Total	*p* *
^†^ My risk of breast cancer				
In the succeeding 5 years	3 (2–3)	3 (2–3)	3 (2–3)	0.16
Lifetime	3 (2–3)	3 (3–4)	3 (2–3)	0.08
^†^ My risk of breast cancer in comparison to BC risk of women of the same age				
In the succeeding 5 years	3 (2–3)	3 (2–3)	3 (2–3)	0.34
Lifetime	3 (2–3)	3 (3–4)	3 (2–3)	0.16
** Breast cancer worry				
In the succeeding 5 years	3 (1–3)	3 (2– 4)	3 (2–4)	0.16
Lifetime	3 (2–4)	3 (2–4)	3 (2–4)	0.18
	**All Other Women *n* = 190**	**Informed Women Aware of Their High BD (*n* = 21)**	**Total (*n* = 211)**	*p* *
^†^ My risk of breast cancer				
In the succeeding 5 years	3 (2–3)	3 (2–4)	3 (2–3)	0.16
Lifetime	3 (2–3)	3 (3–4)	3 (2–3)	0.07
^†^ My risk of breast cancer in comparison to BC risk of women of the same age				
In the succeeding 5 years	3 (2–3)	3 (2–4)	3 (2–3)	0.17
Lifetime	3 (2–3)	3 (3–4)	3 (2–3)	0.03
** Breast cancer worry				
In the succeeding 5 years	3 (1.25–4)	3 (1.5–3)	3 (1.5–3)	0.64
Lifetime	3 (2–4)	3 (2–3.75)	3 (2–4)	0.79

* Mann–Whitney *U* test: data are presented as medians (IQR 25–75%) of grades 1 to 5; ^†^ 1 = very low, 2 = low, 3 = average, 4 = high, 5 = very high; ** 1 = I am not worried at all; 2 = my worries about BC are small; 3 = my worries about BC are moderate; 4 = my worries about BC are big; 5 = my worries about BC are very big.
